# Variations in COVID-19 Spread and Control Measures in the Palestinian Territories

**DOI:** 10.3389/fpubh.2021.736005

**Published:** 2021-08-25

**Authors:** Yehia Abed, Amira Shaheen, Ali Abedrabbo

**Affiliations:** ^1^School of Public Health, Al-Quds University, Jerusalem, Palestine; ^2^Juzoor, Gaza, Palestine; ^3^Faculty of Medicine & Health Sciences, An-Najah National University, Nablus, Palestine; ^4^DG in MOH, Ramallah, Palestine

**Keywords:** COVID-19, control measures, Gaza Strip, East-Jerusalem, West Bank—Palestine

## Abstract

Palestinians are facing the epidemic while they are the only occupied country globally, with around 2 million inhabitants under siege in the Gaza Strip (GS) for the last 14 years and have no control over the health of the Palestinians in East-Jerusalem (EJ). Such catastrophic situations created a variety in the spread of the COVID-19 pandemic in different territories. This study aimed to explore variation in COVID-19 spread, risk factors, and intervention activities in the three Palestinian territories: West Bank (WB), EJ, and GS to learn from the current gaps to overcome this pandemic and be prepared for future emergencies. Epidemiological data regarding COVID-19 were obtained from online websites, Palestinian national reports, WHO reports, and scientific publications. Morbidity and mortality indicators in Palestine are higher than the global level with rate variation in the three territories. COVID-19 incidence and mortality rates are higher in EJ and lowest in GS, while case fatalities are around 1% all over the country. Social gathering and lack of readiness of the fragmented health systems (there are two systems; Palestinian serves the WB and GS and Israeli serves the EJ) are risk factors in the three Palestinian territories. The most prominent risk in GS is overcrowding, while the movement of the workers inside Israel and travel are more prevalent in the WB and EJ. The WHO and international organizations play an active role in responding to a community spread, mainly national coordination, risk communication and community engagement, laboratory support, surveillance and procurement, and supply management. Recommendations include restructuring the national committees, reviewing and standardization of the national protocols, expanding infections prevention training, supporting and developing the capacity of laboratories, and setting the role of NGOs besides community engagement and participation.

## Introduction

Palestinian territories are located on the eastern coast of the Mediterranean Sea. The remaining area from Palestine is divided into two geographically distinct regional units, the West Bank (WB) and the Gaza Strip (GS), while East-Jerusalem (EJ) and areas classified as “C” are under the control of Israel. According to the Palestinian Central Bureau of Statistics, the total population in 2017 was about five million, among them two million throughout the last 14 years locked in the Gaza Strip (GS). With its 365 km^2^, the GS is one of the most densely populated areas in the world (5,324 people per square kilometer) to be 10-fold higher than the WB population density (1). In one of the United Nations Relief and Works Agency for Palestine Refugees in the Near East (UNRWA) camps, population density reaches over 80,000 per square kilometer (2).

Palestinians are facing the pandemic while they are the only occupied country in the world and have no control for the health of the Palestinians in EJ. Such catastrophic situations created a variety in the spread of the COVID-19 pandemic in the different territories. In this study, we tried to demonstrate the fight of the Palestinians to ensure control of the pandemic under challenging circumstances.

In December 2019, a respiratory coronavirus emerged from a large metropolitan area in China's Hubei province, Wuhan. Most cases present with fever, dry cough, and tiredness, although clinical presentation ranges from asymptomatic to atypical severe pneumonia (3). By March 11, 2020, the WHO declared the COVID-19 pandemic (4). Preventive measures are the only solution to save lives and provide the countries with more time to prepare for the arrival of the virus (5). However, within a short time, the disease spread to include most of the countries in the world. In Palestine, the first case was reported in Bethlehem city in the WB on March 6, 2020. The effort focused on the complete isolation of the town and the closure of markets, schools, universities, mosques, churches, and a ban of major social gatherings. In Gaza, the first two cases appeared 3 weeks later than in the WB (March 26, 2020), which allowed time to prepare the same regulations.

This study aimed to explore the variation in COVID-19 spread, risk factors, and intervention activities in the three Palestinian territories: West Bank, East-Jerusalem, and Gaza Strip to learn from current gaps, to overcome the current pandemic, and face future emergencies.

## Study Objectives

1. To explore differences in morbidity and mortality indicators in the Palestinian territories,
2. To reveal differences in risk factors for the COVID-19 spread in the three territories,
3. To compare the response to the pandemic in the Palestinian territories,
4. To formulate recommendations to policymakers to overcome the pandemic.

## Methodology

Epidemiological data regarding COVID-19 were obtained from online websites, mainly from the Palestine COVID-19 website (https://www.corona.ps/details) and the Worldometer for coronavirus (https://www.worldometers.info/coronavirus/), and from the Palestinian national reports, WHO reports, and scientific publications. The research team reviewed these data, and the following rates were calculated: the cumulative incidence and COVID-19-specific mortality and case–fatality rates (CFRs). In the end, we compared the reported data in the three study territories.

We abstracted the morbidity rate by calculation of the cumulative incidence rate per 1,000 inhabitants by dividing the cumulative number of the reported cases divided by the total population of the country multiplied by 1,000. Two indicators were calculated for mortality, first was the COVID-19-specific mortality rate calculated by the total number of COVID-19 deaths in the country divided by the total population of the country and multiplied by 100,000. The second mortality indicator is the COVID-19 case–fatality percentage and calculated by dividing all cases of COVID-19 deaths by the reported number of reported cases and multiplied by 100. Covid mortality reporting was based on COVID-19-positive testing and the death certificates reporting following the COVID-19 guidelines of the WHO for death certification and coding (ref). Rates were calculated based on the Palestinian Central Bureau of Statistics (PCBS) estimate of the Palestinian population (WBG) (5,227,193) in the WB (3,120,448), GS (2,106,745), and EJ ID holders (428,304) (6).

For calculation of the risk factors, crowding was calculated by the population density as measured by the number of the population per square kilometer while travel limitation for Gaza population based on the WHO and The UN Office for the Coordination of Humanitarian Affairs (OCHA) reports. Readiness of health services was based on the MOH and international reports. Responsiveness to the COVID-19 pandemic is based on comparing MOH reports with the WHO components to respond to the community spread of COVID-19 (7, 8).

## Results

The study results are presented in three major components, and each part shows the variation among the three Palestinian communities in:

1. COVID-19 morbidity and mortality.
2. Risk factors for COVID-19.
3. Responsiveness to the COVID-19 pandemic.

## Variation in COVID-19 Morbidity and Mortality

Table 1 shows the cumulative incidence rate per 1,000 inhabitants, COVID-19-specific mortality rate per 100,000 inhabitants, and case fatality as a proportion of deaths from all the COVID-19-confirmed cases in the Palestinian territories and selected countries.

**Table 1:** COVID-19 in selected countries, mid-March 2021.

**Country**	**Cases/1,000**	**Deaths/100,000**	**Case**	**Tests/1,000**
			**fatality %**	
World	15	34		
United States	98	162	1.65	1,114
Israel	87	64	0.73	1,357
United Kingdom	62	183	2.95	1,416
Turkey	33	34	1.03	403
Egypt	2	11	5.50	10
Jordan	42	48	1.14	478
Lebanon	58	75	1.30	467
Palestine	**41**	**43**	**1.06**	**248**
East-Jerusalem	63	55	0.87	
West Bank	47	53	1.12	
Gaza Strip	27	26	0.98	191

*Rates were calculated based on the PCBS estimate of the Palestinian population (WBG) (5,227,193) in the West Bank (3,120,448), Gaza Strip (2,106,745), and East-Jerusalem ID holders (428,304) (6). Data were abstracted from https://www.corona.ps/details, https://www.worldometers.info/world-population/state-of-palestine-population (https://www.worldometers.info/coronavirus/)*.

The reported COVID-19 morbidity and mortality is acceptable compared with those of the other countries but higher than the global average. The cumulative incidence rate for COVID-19 is 41 cases per 1,000 inhabitants, 2.7-folds more elevated than the global cumulative incidence of 15 and similar to Jordan with 42 but less than Lebanon with 58. Countries such as the United States, Israel, and the United Kingdom reported higher incidences, possibly due to massive community screening where tests in these countries exceeded the number of the population. In contrast, Palestinians reported a low rate of tests completed per 1,000 inhabitants: 238 tests per 1,000). Two mortality indicators are considered: COVID-19-specific mortality rate per 100,000 and case fatality as a percentage. The first mortality indicator shows that the COVID-19-specific mortality rate in Palestine with 43 is higher than the global rate of 34 and similar to the Jordanian rate but less than those of Lebanon, Israel, and the United States. Case fatality reflects the severity of the disease and the response and capability of the health services to manage the reported cases. In Palestine the CFR is around one, a little higher than that of Israel but lower than those of Lebanon and Jordan. The lowest case fatality was reported in GS as shown in Table 1.

By mid-March 2021, the reported cumulative incidence was 41 per thousand inhabitants with variation between the territories, the highest in EJ with 63 per thousand and the lowest was in the GS with 27 per thousand while WB reported 47 cases per thousand inhabitants. This variation in the reported cases between the three territories was due to the low traffic movement in the GS because of continuous closure of the borders, specifically Rafah. The GS saved 7 months with zero community transmission due to the restrictive measures of 21 days isolation of all travelers coming to Gaza at the beginning of the pandemic. The situation was different in EJ and WB: transmission of new strains of the virus between Israel and the WB was enhanced by the 180,000 Palestinians working inside the Greenline and Palestinians holding EJ and Israeli IDs. In addition, there is no security control; hence it was difficult to control the pandemic. More than 800 cases were reported in the WB from English and South African strains.

The differences in the cumulative incidence affected the specific mortality rate per 100,000 inhabitants to be highest in EJ (55) followed by the WB (53) and the lowest rate reported in the GS (26), the total specific mortality rate for Palestine to be 43 per 100,000 inhabitants. Despite the variation of COVID-19-specific mortality rate in the Palestinian territories, CFR at Eastern Mediterranean Region reached 2.37% on January 30, 2021. The highest was in Yemen (29%) (9). In Palestine, the case fatality was around 1%, with the lowest in EJ (0.87).

## Variation in risk factors

Studies showed common risk factors for spreading the disease in different countries, including overcrowding, travel between countries, social gathering, availability of health services, and the response of the health authority to the event. Table 2 shows a summary of the variation of risk factors for COVID-19 for the three territories.

**Table 2:** Summary of the variation of risk factors for COVID-19 for the three territories.

**Risk**	**Gaza**	**West Bank**	**E. Jerusalem**
1.Overcrowdness Population density	++++++	++	+++
	5,324 persons per km^2^	^*^509 persons per km^2^	
2.Travel and movement	+	++	+++
3.Worker risk	+	++	+++
4.Social gathering	+++	+++	++
5.Health readiness shortage of	+++	++	+
A. Diagnostic facilities	+	++	+++
Intensive care beds	+	++	+++
Ventilators devices per 100,000	4	10	
O_2_ Generators	++	++	++
Medications	+	++	+++

* *https://www.pcbs.gov.ps/Portals/_Rainbow/Documents/Land-use-table%201E-2019.html*.

### Overcrowding

Overcrowding is a known risk factor giving the chance for spread among the population. In the GS, the population density is among the highest globally, with 5,324 inhabitants per square kilometer. Thus, the population density in the GS is 10 times higher than that in the WB, keeping the population in GS at an increased risk of the rapid spread of the infection. Yet, the opposite happened as described above.

### Travel and Movement

There are marked differences between the Palestinian territories in control of the borders, either at entry or exit to the GS, closed during the last 14 years. This situation created geographically and security-wise a status different from the WB and resulted in limited movement between the GS and the external world. The crossing areas of the GS are controlled at only two points, the Beit

Hanon (Eretz) crossing for those coming through Israel and the Rafah crossing for those coming through Egypt. This negatively affected the socioeconomic status of the population and imposed significant obstacles in patient referrals outside the GS. It also created substantial barriers in drugs, medical supply, and equipment to the GS. For the WB, the crossings with Israel faced problems due to the diversity of crossing points and the loss of security control by the Palestinian side. In contrast, EJ and areas “C” are entirely controlled by Israel. As a result, many Palestinian workers from the WB pass through these crossings and constitute a high risk of virus transmission from Israel to the Palestinian territories. During the early stages of the pandemic, 75% of the positive cases recorded in the Palestinian territories were workers inside Israel and their contacts. The same applies to the border with Jordan, which remained open for a while.

### Social Gathering

Social gathering is typical in all the Palestinian territories without variation; wedding parties and funerals were the most common factors in spreading the disease in the Palestinian territories. The same applied to educational institutions, market gathering, and general transport with limited precautions. In addition, social groups included religious places such as mosques and churches, especially on Fridays for Muslims and Sundays for Christians.

### Readiness of Health Services

Political insecurity and socioeconomic instability have affected the health of the population and the ability of Palestinians to develop a modern health system, particularly intensive care rooms, respirators, and lack of access to serve residents in the neighborhoods of EJ and the occupied areas “C” in the WB. The respirator rate in Palestine is 10 devices per 100,000 citizens and 4 in the GS. Compared to other countries, these rates are 30 in Germany and 50 in Israel, while Israelis seek to raise them to 150 devices per 100,000 inhabitants (10). The impact of the political split has been severe and harms the population of the GS. In addition, there is a chronic shortage of essential medicines and health supplies for more than one-third of what is needed, especially in emergency rooms, operation theaters, intensive care units, orthopedic services, nephrology centers, and neonatal care units. The Palestinian people have faced many restrictions that have affected their ambition to create a functional Palestinian health system during the last 14 years. In East-Jerusalem isolation and restriction is a major factor as residents are unable to access Israeli hospitals (11).

## Responding to community spread of COVID-19 variation

In this part of the results, we followed the WHO components to respond to the community spread of COVID-19 (7, 8). WHO experts set technical guidance of 10 items to help government authorities, health workers, and key stakeholders to guide response to community spread of the disease. We compared the three Palestinian communities for each of these items.

### National Coordination

There are central committees and regional committees to support coordination, planning, and monitoring. Examples of major committees are the National Epidemiology Committee and the Vaccine Purchase Committee. These committees were formed with EJ, WB, and GS and chaired by the Minister of Health. At the same time, there are committees in both WB and Gaza to support the authorities in planning and surveillance of events. These committees are advisory committees to respond to the situation based on the variation of case occurrence and the health facilities available in each Palestinian territory. However, they often lack efficient coordination. In most instances, WHO, UNICEF, and the National Institute of Public Health are also present in regional committees and play a significant role in standardizing plans and monitoring.

### Risk Communication and Community Engagement

The WHO supports this area through Health Cluster meetings: international and local organizations monitor risk communication and community engagement. All the results are shared with the Palestinian side (12). One example is this site where all materials are categorized under one of these groups: general advice, hand washing, hygiene, quarantine and isolation, COVID-19 vaccine, education, face masks, family care, gender-based violence, nutrition and exercise, physical distance, safe shopping, stigma and discrimination, and the workplace. Each category of the above contains videos, health messages, social media cards, radio spots, and brochures. Most of them are published on Facebook, Instagram, and Twitter (see example in the references further). Modern technology makes all material available to all Palestinian sites (13, 14).

### Public Health Measures

There are variations in the Palestinian territories in public health measures due to variation in time of community infection, available facilities, and controlling authorities. Table 3 shows variations and similarities in the different localities.

**Table 3:** Summary of the interventions for COVID-19 for the three territories.

**Intervention**	**Gaza**	**West Bank**	**E. Jerusalem**
Date of new cases discovery	Among Quarantines: March 26, 2020 Community cases: August 16, 2020	March 6, 2020	March 23, 2020
Quarantine Measures	All people enter Gaza 21 days starting March 15, 2020	Starting March 6 to May 26	
Quarantine period	21 days	14 days	14 days
Closure and Governorate separation	Once August 26, 2020	First time March 6, 2020	March 19 to May 4; July 6 to October 18; and again, on December 24 to January 9^*^
Night closures + Saturday and Sunday	Nov 15, 2020 to February 4, 2021	During the summer and again in November and December	On September 6, the Israeli government approved night-time curfew
Chances of vaccination^**^	Expected to have 40% of all vaccines arriving Palestine	120,000 workers inside Israel had the chance to get the Vaccine	Have better chances for vaccination within the Israeli system

* *http://www.wclac.org/files/library/21/03/m8bwzl5xfxczrncl5ygkyi.pdf (16)*.

** *Recent immunization data are available on: www.emro.who.int/pse/palestine-infocus/situation-reports.html*.

The first case was reported in Bethlehem city in the WB on March 6, 2020, the government efforts focused on the complete isolation of the town and the closure of markets, schools, universities, mosques, churches, and a ban of major social gatherings. As for the GS, since March 15, 2020, all travelers, coming in through one of the two crossing points, were transferred to a compulsory quarantine at one of the MOH designated facilities (15). The quarantine period exceeded the 14 days recommended by the WHO by an extra week to avoid possible incubation periods longer than 2 weeks. In Gaza, the first two cases appeared 3 weeks later than in the WB (March 26, 2020), which allowed time to prepare the same regulations. Nevertheless, the community spread of the virus was first reported in GS by August 16, 2020. As a result, immediate and vigorous lockdown for the entrance points was implemented immediately, followed by gradual canceling of the compulsory quarantine and replaced by a medical check-up and PCR testing. Likewise, the Palestinian authorities declared emergency status for WB by the day of discovering the first case. They also called for the closure of educational and religious facilities and ordered restrictions on gatherings and movement between cities. The first lockdown lasted from March to May 26, 2020, but was soon followed by further restriction measures. Subsequent lockdowns resulted in a reduction of registered cases. Palestinians in EJ are subject to Israeli public health regulations besides lockdown with closures and restrictions for three cycles (16).

### Case Management and Health Services

The blockade imposed on the GS deprived the developmental activities and prevented the entry of equipment, medicines, and diagnostic materials, deteriorating the diagnostic and treatment facilities. The technical committee developed protocols to ensure proper diagnosis and management of COVID-19 (17). Those protocols were based on the WHO publications and the frequent modifications (18). Unfortunately, those committees worked separately in WB and GS despite the personal communications between the scientists on both sides and participation in zoom meetings in training sessions with international conferences. Currently, there are two versions of protocols, one for the WB and one for the GS. Training sessions were completed through in-service training for the health staff. The training covered infection prevention, diagnostics, oxygen therapy, antimicrobials, mechanical ventilation, sedation, best practices to prevent complications, liberation from mechanical ventilation, quality in critical care, pandemic preparedness, and ethical considerations. The training was organized by MOH/Human Resource Education or through funded activities by international organizations, national and international NGOs, and electronic communication with experts from outside the country. The recent zoom meetings gave equal chances for experts from GS, EJ, and WB to meet and share the learning experience with international experts. These programs reached about approximately 2,000 health care workers.

### Infection Prevention and Control

Prevention of infection was planned at three levels: first, support of health education activities focusing on the importance of wearing face masks, hands wash, and surface disinfection. Second, closing overcrowded places such as schools, universities, mosques, sports clubs, wedding halls, funeral homes, and major markets. Third, isolation of communities with confirmed cases, either by curfew or restriction of movements between communities. In the early stages of the pandemic, all three levels were implemented in the WB, while in the GS activities were limited to the first two levels, sufficient to delay in the GS community transmission for 7 months. Finally, all health staff were trained for rational use of personal protective equipment and infection prevention. Training covered especially the following topics: IPC, infection transmission, hand and respiratory hygiene, injection safety, decontamination, environmental cleaning, and waste management. Despite these efforts, Gaza reported 437 COVID-19 cases among the health workforces during 1 week based on the local MOH report dated April 17, 2021.

### Surveillance and Risk and Severity Assessments

Palestine adopted the WHO case definition for COVID-19 and issued a daily report for GS, WB, EJ, and Palestinians in diaspora (https://corona.ps/). The report contained some new cases in each governorate, the cumulative number of cases, active cases, cured cases, and total deaths. In addition, curves demonstrated daily changes in the related indicators. Palestine also participated in the WHO serosurvey for COVID-19 in WB and GS. Recently, in addition, a surveillance system of vaccination activities was established, which allows collecting data on vaccine types and complications.

### National Laboratory Systems

Facing the current pandemic requires the preparation of laboratory facilities to respond to a vast number of sampling testing for diagnostic, follow up, and surveillance purposes, besides laboratory facilities for blood, urine, and stool samples of patients. The major problem faced by the MOH is the availability of PCR testing where machines and swabs were not available. WHO suggested the support of the National Public Health Laboratory in Ramallah/WB to carry out PCR testing for both the WB and the GS. However, WHO supported GS Lab with a PCR machine and swabs as it turned out not to be easy to send the GS samples to WB. Under the control of MOH, permission was given to NGOs to conduct the COVID-19 PCR testing. Protocols were developed on how to collect, transport, and examine the samples. The epidemiology and laboratory teams were trained accordingly. With the spread of different COVID-19 strains to the WB and GS, special kits posed additional costs.

### Logistics, Procurement, and Supply Management

The Health Cluster Committee (chaired by the WHO and the MOH) calls for international sources and regulates the distribution in WB and EJ. In addition, direct purchase in Ramallah and Gaza is made to face urgent requests. At the same time, direct donations for GS or WB are registered in the main stores and distributed based on need. A good example is also the establishment of the National Committee for Vaccine Purchase.

### Maintenance of Essential Services

In the early days of the pandemic (March 6, 2020), several Primary Health Care (PHC) centers were closed in both GS and WB to give support to hospitals in preparation and epidemiological investigations. WB health centers suffered more due to staff barriers to reach their working places. However, based on the WHO recommendations, essential health services should continue during emergencies, particularly immunization programs, antenatal care services, and care of chronic patients (19). During the first 3 months, UNRWA provided most of the PHC activities and established a public hotline to serve people with home treatment and health consultations, ready to reach all those registered for non- communicable disease services. Also, social assistance at home was secured to avoid overcrowding in the centers. Furthermore, UNRWA offered vaccination programs for children. Later, starting from August the first wave started to decline in the WB and started to increase in Gaza and the centers were gradually reopened in most of the territories.

Health Cluster Committee continues to monitor the status of service provision in the Palestinian localities. For example, they stated that: “In Gaza, 27 (84%) fully functioning hospitals, 5 (16%) partially functioning hospitals, 135 (91%) functioning primary health care clinics, 5 (3%) partially functioning primary health care clinics, 9 (6%) not functional.” Also, in the same report, with regard to NGOs providing services through mobile teams in closed areas and areas facing a shortage of health staff in the WB: “8 mobile medical teams/clinics currently provide primary health care services.” All partners continued efforts to ensure continued access to essential services, such as primary healthcare, sexual and reproductive health, surgical care, nutrition, as well as mental health and psychosocial support (19).

### Research and Development

Palestinian researchers participated in some local and international research studies. Members from Palestinian universities in WB, GS, and EJ published papers about the prevalence of risk factors for COVID-19, satisfaction with the services, etc.

Knowledge, attitude, and practice studies among the population about the pandemic and perceptions of health care workers regarding local infection prevention and control procedures. PCBS, MASS, MASARAT, PINGO, and others published relevant reports. The American Arab University in Jenin completed genotyping studies. Juzoor University completed a study on the impact of the COVID-19 outbreak and lockdown on family dynamics and violence (20). The Palestinian Public Health Institute participated in the WHO sero-surveillance study. Studies are ongoing on the impact of COVID-19 on health services. Palestinian members in the scientific committee follow recent studies for COVID-19, searching for evidence of decision making and development of protocols and guidelines. It is worth to note that most of the studies are social and simple epidemiological studies due to the absence of research laboratory infrastructures in Palestine.

## Discussion

Globally, most countries experienced facing COVID-19 pandemic with the difference in the severity of spread, complications, risk factors, and variation in response mechanisms. Palestinians faced the pandemic under difficult political situation presented by a political division between the GS and the WB beside uncontrolled for parts in the WB and EJ. Our findings showed differences between the Palestinian territories where, the COVID-19 incidence and mortality rates were higher in EJ and lowest in GS, while case fatality was around 1% of all cases all over the country with minor variation between the three localities. However, we expected a higher incidence in the GS because of the 10-fold higher population density, which did not happen either due to political blockade on Gaza preventing the movement of travelers or due to under-reporting of cases. The reported COVID-19-specific mortality rate of Palestine was 43 per 100,000 was higher than the global rate of 34 per 100,000 and similar to the Jordanian rate but less than those of Lebanon, Israel, and the United States. The high reported rates in the United States of 162 and England with 183 can be explained by a high proportion of the aged population with higher risk for COVID-19 complications. Higher case fatalities in the United States and England reflect the severity of cases among the aged population and could be attributed to different strains. Anyhow, mortality comparison between countries is no easy task because of the differences in population structure and inaccuracy of diagnosis of the cause of the death and real implementation of the WHO guidelines in practice within countries (21, 22).

This study showed variation in risk factors between the three territories. The most prominent risk in GS was overcrowding, while the movement of the workers inside Israel and travel is more prevalent in WB and EJ. The social gathering was one of the major risk factors for reporting a cluster of cases among the Palestinian population. In the GS, where there is a low traffic movement, despite having the highest population density, it was the lowest cumulative incidence and the lowest COVID-19-specific mortality, among the three Palestinian territories. Besides, Gaza followed quarantine measures—all travelers coming to Gaza in the first 7 months of the pandemic were placed in a 21 days isolation quarantine. During the 7 months, the GS with the support of the WHO and donor community got a chance to prepare the health care system by improving the laboratory facilities, increasing the number of intensive care units, oxygen generators, personal protective devices, and training of the staff.

Variation in response mechanisms to the COVID-19 pandemic is associated with the unique political situation that created a complex environment with major obstacles blocking short-term solutions for the current health problems and preparedness for expected epidemics. Overcrowding,' movement of workers, and change of the population social gatherings are not easy risk factors to overcome. At the same time, political changes by removing border restrictions and ending the occupation although that it is a necessity but in reality, no active steps are seen soon. Based on that the authors set their recommendations that could be applied to minimize the current health hazards and to enable the health care system to face the current pandemic and future undesirable events based on the current COVID-19 experience.

During the current COVID-19 pandemic, WHO played an active role in preparedness and response to face the pandemic in Palestine. Coordination through Health Cluster group made fare distribution of the international support by donor communities that enabled Palestinians to support the emergencies in the three territories. The study showed the importance of the formulation of the National Committees and the regional subcommittees and their major role to coordinate different activities to ensure proper surveillance system and fair distribution of the available resources and follow the international scientific regulations to standardize the control measures all over the Palestinian land. Following the WHO guidelines to respond to the COVID-19 pandemic helped the Palestinians to develop their capacities to face the pandemic. National committees have been formed which meet and exchange information and scientific documents regularly. The committees recommended public health measurements implemented based on the situation in each territory. The technical committee succeeded to develop protocols for case management based on the WHO guidelines, which require further modifications to ensure the use of one National protocol based on the COVID-19 experience. Palestinians have to benefit from the health cluster experience to support coordination with the international and donor communities and based on the experience of the current national committees, restructuring of the different scientific committees will minimize the variations between the territories and improve communication and future interventions.

The pandemic revealed that there is a major gap in the health care system toward infection prevention as a high number of infected health workforces remain as a public health problem despite training courses and the provision of personal protective devices.

By Palestinian Public Health law (23), control of the epidemics is the responsibility of the Government and all services are free from fees. One major achievement during the pandemic is the maintenance of essential health services such as basic immunization program, Ante Natal Care, and Non-Communicable Diseases management. That was not possible to achieve without full cooperation with the other health care providers. Mainly UNRWA, NGOs, and the private sector. UNRWA supported the provision of PHC services and later prepared their centers to be ready for population vaccination. NGOs reached isolated communities, provided PHC services and elective surgeries besides their role in supporting risk communication and community engagement activities. This experience guided the health policymakers to reset the role of the different health care providers during a normal situation and during emergency time.

The time of the pandemic was not easy for the Palestinians and all the world countries, but we have to learn from all these lessons how to improve the health care system and to be ready for any future pandemics.

## Recommendations

1. Restructure of the national committees to be one central and two regional sub-committees, one for EJ and WB and one for GS, and to generalize this structure for different national committees to ensure standardization of the Palestinian health care system.
2. Review and standardize all national protocols, guidelines, and curricula for training.
3. Expansion of infections prevention training to all health care facilities and health-related higher education faculties.
4. Support and development of laboratory capacity in both WB and GS to ensure the capability to face emergencies.
5. Setting the role of NGOs during a normal situation and during emergency time.
6. Support of the research infrastructure to help Palestinian researchers to meet the community needs and contribute to an international network.
7. Community involvement and participation to support the official authority in the field to implement their plans and activities. However, clearly defined tasks are needed.

## Data Availability Statement

The original contributions presented in the study are included in the article/supplementary material, further inquiries can be directed to the corresponding author/s.

## Author Contributions

YA gathered the data and analyzed and drafted the text. AS provided East Jerusalem Data and revised the text. AA matched the data with the MOH reports and revised the text. All authors contributed to the article and approved the submitted version.

## Conflict of Interest

The authors declare that the research was conducted in the absence of any commercial or financial relationships that could be construed as a potential conflict of interest.

## Publisher's Note

All claims expressed in this article are solely those of the authors and do not necessarily represent those of their affiliated organizations, or those of the publisher, the editors and the reviewers. Any product that may be evaluated in this article, or claim that may be made by its manufacturer, is not guaranteed or endorsed by the publisher.
